# A CRISPR screen for factors regulating SAMHD1 degradation identifies IFITMs as potent inhibitors of lentiviral particle delivery

**DOI:** 10.1186/s12977-018-0409-2

**Published:** 2018-03-20

**Authors:** Ferdinand Roesch, Molly OhAinle, Michael Emerman

**Affiliations:** Divisions of Human Biology and Basic Sciences, Fred Hutchinson Cancer Research Center, 1100 Fairview Ave N, Mailstop C2-023, Seattle, WA 98109 USA

**Keywords:** HIV-1, SAMHD1, Vpx, Pseudotypes, Interferon, IFITM, VSV-G, A-MLV, Viral entry

## Abstract

The InterFeron Induced TransMembrane (IFITM) proteins are interferon stimulated genes that restrict many viruses, including HIV-1. SAMHD1 is another restriction factor blocking replication of HIV-1 and other viruses. Some lentiviruses evolved Vpx/Vpr proteins to degrade SAMHD1. However, this viral antagonism can be perturbed by host mechanisms: a recent study showed that in interferon (IFN) treated THP1 cells, Vpx is unable to degrade SAMHD1. In the present work, we designed an Interferon Stimulated Genes (ISGs)-targeted CRISPR knockout screen in order to identify ISGs regulating this phenotype. We found that IFITM proteins contribute to the IFNα-mediated protection of SAMHD1 by blocking VSV-G-mediated entry of the lentiviral particles delivering Vpx. Consistent with this, IFNα treatment and IFITM expression had no effect when the A-MLV envelope was used for pseudotyping. Using an assay measuring viral entry, we show that IFNα and IFITMs directly block the delivery of Vpx into cells by inhibiting VSV-G viral fusion. Strikingly, the VSV-G envelope was significantly more sensitive to this IFNα entry block and to IFITMs than HIV-1’s natural envelope. This highlights important differences between VSV-G pseudotyped and wild-type HIV-1, in particular relative to the pathways they use for viral entry, suggesting that HIV-1 may have evolved to escape restriction factors blocking entry.

**Electronic supplementary material:**

The online version of this article (10.1186/s12977-018-0409-2) contains supplementary material, which is available to authorized users.

## Background

Type I IFNs play a central role in activation of innate immunity by turning on a vast transcriptional program that results in enhanced expression of hundreds of Interferon Stimulated Genes (ISGs). Many of these ISGs encode proteins that have antiviral activity, inhibiting viruses at various steps of their life cycle including at the earliest stages of entry [1]. In particular, the InterFeron Induced TransMembrane (IFITM) proteins (IFITM1, IFITM2, and IFITM3) are ISGs localized at the cell surface and in endosomal compartments that restrict entry of many viruses, including HIV-1, VSV, Influenza A, some flaviviruses and alphaviruses [2–6].

After fusion, retroviruses release genomes and associated proteins into the cytoplasm during the uncoating process, ultimately resulting in the synthesis of reverse transcription products, formation of the preintegration complex, and nuclear import [7]. The accessory proteins Vpr and Vpx, which are encapsidated into budding virions through interaction with the p6 region of Gag [8], are also released from the incoming viral particles after fusion, in a process independent from uncoating of the viral core [9].

One of the functions of Vpx is to promote degradation of the host antiviral protein SAMHD1 [10, 11]. SAMHD1 is a dNTP triphosphohydrolase that depletes the cellular pools of dNTPs [12] in quiescent cells of the myeloid lineage and in resting CD4 + T cells [13], resulting in a block to reverse transcription [10, 11]. In some SIVs lacking Vpx, SAMHD1 antagonism is achieved by the related viral protein Vpr [14]. Vpx/Vpr proteins directly bind to SAMHD1 and recruit the DDB1/Cul4/DCAF1 ubiquitin ligase complex, resulting in proteasomal degradation of SAMHD1 [15, 16]. The recognition of SAMHD1 by Vpx/Vpr proteins is evolutionarily dynamic, with some viral proteins binding to the N-terminus of SAMHD1, and others to the C-terminus [17]. In addition to the evolution at the Vpx/SAMHD1 interface, the host has evolved other mechanisms to regulate SAMHD1. For instance, SAMHD1 enzymatic activity is controlled by cell cycle progression [18–20], and the host factor H11/HSPB8 has been reported to degrade Vpx, thus protecting SAMHD1 from degradation [21].

Dragin et al. [22] showed that interferon alpha (IFNα) treatment of THP1 cells prevents degradation of SAMHD1 following incubation with SIV_MAC_ virus-like particles containing Vpx (VLPs-Vpx). This observation suggests the existence of one or more proteins induced or activated by IFNα that directly or indirectly protect SAMHD1 from degradation. IFN treatment controls SAMHD1 enzymatic activity by phosphorylation [18], and reports have described induction of SAMHD1 expression in monocytes and some cell lines after IFNα treatment [23, 24] through mechanisms involving modulation of microRNAs and IRF3, respectively. However, none of these effects could explain how IFNα protects SAMHD1 from degradation by Vpx [22].

In the present work, we confirmed that treatment of THP1 cells with either interferon alpha (IFNα) or interferon gamma (IFNγ) indeed protects SAMHD1 from degradation by Vpx when Vpx is delivered to cells via VLPs that are pseudotyped with VSV-G. We designed a flow cytometry-based CRISPR knockout screen to identify ISGs involved in this phenotype and identified the InterFeron Induced TransMembrane (IFITM) proteins. Moreover, we show that IFNα protects SAMHD1 from degradation when Vpx-containing VLPs are pseudotyped with VSV-G for viral entry, but not when they are pseudotyped with the envelope from amphotropic murine leukemia virus (A-MLV), suggesting that the IFNα block occurs at the level of viral entry. By directly comparing the entry block imposed by IFNα and IFITMs on HIV-1 pseudotyped with different envelopes, we show that VSV-G is inhibited to a greater degree than HIV-1 wild-type envelope. This result suggests that HIV-1 may escape IFNα induced blocks to viral entry, potentially by using different cellular pathways for membrane fusion, as suggested in a recent study [25]. Finally, our screen could also be used to identify factors blocking a variety of different viral envelopes, using SAMHD1 degradation as a proxy for cytoplasmic delivery of viral proteins.

## Results

### IFN treatment protects SAMHD1 from degradation by Vpx in THP1 cells

**Fig. 1:**
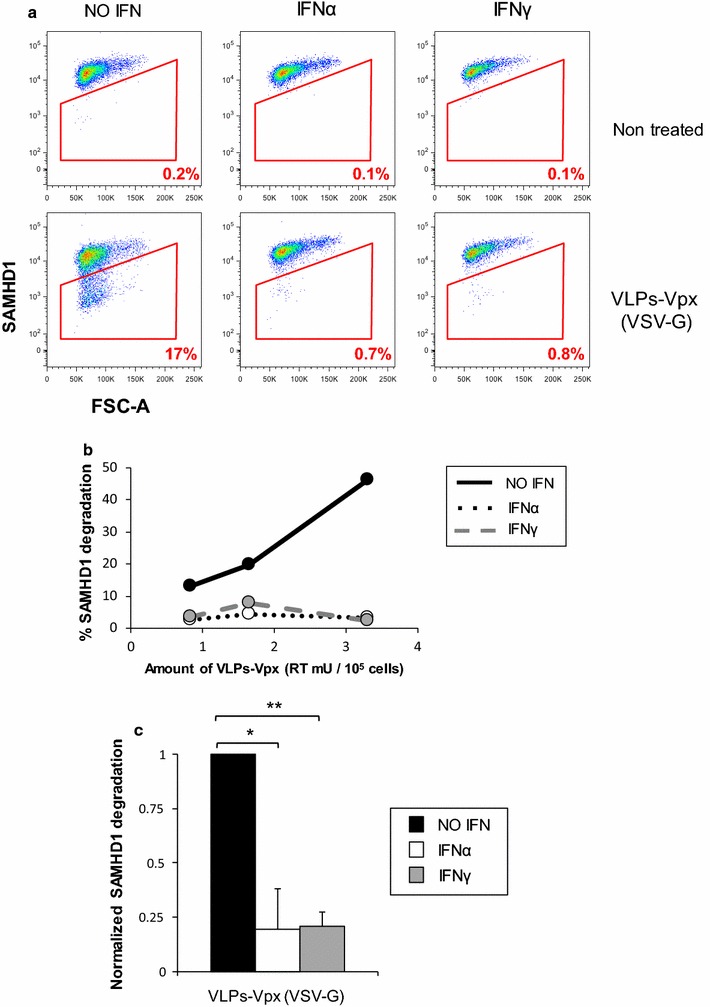
IFN treatment inhibits Vpx-mediated degradation of SAMHD1. THP1 cells were treated for 24 h with 1000 U/mL of IFNα, 1000 U/mL of IFNγ or left untreated. The indicated amount of VLPs-Vpx pseudotyped with VSV-G (as determined by RT activity) was added to cells for 16 h: cells were then collected and SAMHD1 degradation was measured by flow cytometry. **a** Representative flow cytometry pots. **b** Representative dose response experiment. THP1 were treated with the indicated amount of VLPs-Vpx. The percentage of cells in which SAMHD1 is degraded was determined as shown in **a**. **c** Combined data from three independent experiments, using a viral dose within linear range. *p < 0.05; **p < 0.01 (*t* test)

First, we confirmed the published observation that IFN treatment inhibited SAMHD1 degradation in THP1 cells [22]. SAMHD1 degradation in THP1 cells was achieved by overnight treatment with Virus-Like Particles (VLPs) that consist of the SIV_MAC_ Gag/Pol structural proteins and naturally package both Vpr and Vpx [26], with only Vpx being able to degrade SAMHD1 [14]. Vpx-containing VLPs (VLPs-Vpx) were pseudotyped with the Vesicular Stomatitis Virus glycoprotein (VSV-G), as has been done in previous studies [10, 12, 22, 27]. THP1 cells were treated with universal type I interferon alpha (IFNα), a recombinant human IFN alpha, or IFNγ for 24 h and transduced with VLPs-Vpx. We measured the endogenous levels of SAMHD1 using flow cytometry in order to monitor SAMHD1 degradation in a quantitative manner in cell populations. Over 99% of the cells expressed SAMHD1, and this proportion did not change with IFNα or IFNγ treatment (Fig. 1a, top row). Consistent with previously published data, SAMHD1 was degraded in the presence of VLPs-Vpx (Fig. 1a, bottom row) and high levels of SAMHD1 degradation can be achieved: it reached 50% in the dose response presented in Fig. 1b (black lines), and even higher levels if more VLPs-Vpx were used (not shown). However, in the presence of IFNα and IFNγ, SAMHD1 degradation was blocked, (Fig. 1a, bottom row), even at high doses of VLPs-Vpx (Fig. 1b, dashed lines). Averaged over three independent experiments, IFNα or IFNγ treatment blocked SAMHD1 degradation by fivefold (Fig. 1c). Therefore, we hypothesized the existence of one or several ISGs, induced by both IFNα and IFNγ, that protect SAMHD1 from degradation by Vpx-containing VLPs.

### A CRISPR screen identifies IFITMs as factors blocking Vpx-mediated degradation of SAMHD1

IFNα could affect SAMHD1 degradation by Vpx by any of several mechanisms, such as blocking Vpx trafficking to the nucleus, post-translational modification of SAMHD1, interference with the degradation pathway through which Vpx targets SAMHD1, or, as described below, IFNα induction of genes affecting entry/fusion of the VLPs delivering Vpx. In order to identify the factor(s) responsible for this phenotype, we designed a CRISPR knockout screen taking advantage of the high-throughput qualities of both flow cytometry and next-generation sequencing technologies. We hypothesized that sgRNAs that target genes necessary for SAMHD1 protection from degradation would be enriched in the population of cells displaying low levels of SAMHD1. We first created a library of single-guide RNAs (sgRNAs) targeting 1906 human ISGs, with 8 different sgRNAs per gene and 200 non-targeting controls (NTCs) that do not target any loci in the human genome. We assembled sgRNAs into a lentiviral vector backbone that also encodes Cas9 and a puromycin resistance gene (OhAinle et al., manuscript in preparation). THP1 cells were transduced with this library, selected for puromycin resistance and cultured for 2 weeks to allow gene knockout to occur. The cells were treated with IFNα, and incubated with VSV-G pseudotyped VLPs-Vpx as described in Fig. 1. Endogenous SAMHD1 levels were measured and cells were sorted using flow cytometry.

**Fig. 2:**
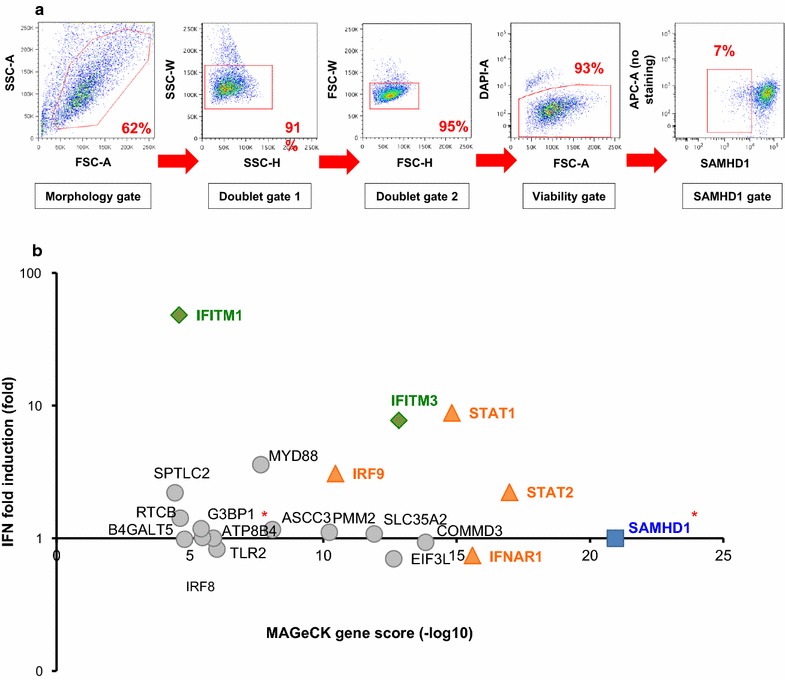
A CRISPR knockout screen identifies IFITMs as blocking SAMHD1 degradation by Vpx upon IFN. α treatment. **a** Sorting strategy. 5 × 10^7^ THP1 cells were treated with 1000 U/mL IFNα for 24 h and then incubated with 2.5 RT units of VLPs-Vpx pseudotyped with VSV-G for 16 h. Cells were harvested, incubated for 30 min with a viability dye, gently fixed with 1% PFA, permeabilized and stained for SAMHD1 as described before. SAMHD1 negative and SAMHD1 positive cells were sorted by flow cytometry on a BD FACS Aria-II using the indicated gates. The FSC/SSC gate allowed to sort out dead cells and debris, based on morphology. The doublet gates 1 and 2 allowed to remove cell doublets, based on height (H) and width (W) for the FSC and SSC parameters. The viability gate allowed to remove dying cells, that fluoresce in the DAPI channel and that exhibit aberant SAMHD1 staining. Cells were sorted on their levels of endogenous SAMHD1. The cutoff used for SAMHD1 negative cells is indicated in the red gate. 5 × 10^5^ SAMHD1 negative, and 3 × 10^6^ SAMHD1 positive cells were sorted to ensure sufficient library coverage. Two technical replicates were performed—one representative flow cytometry plot is shown. **b** Top 20 hits. sgRNA enrichment in SAMHD1 negative cells was determined after Illumina sequencing, and MAGeCK gene analysis was performed to take into account data from all 8 sgRNAs per gene and from two replicate experiments. The MAGeCK score (−log10) of the top 20 enriched genes is indicated on the X axis. Published IFNα induction data in THP1 cells [29] is shown on the Y axis (log scale). For some genes, expression was measured using multiple probes: in that case, we averaged the results. Two genes on our list, SAMHD1 and ATP8B4 (indicated with red asterisks), were absent from the dataset, and were arbitrarily set at 1. SAMHD1 is indicated with a blue square, positive controls, i.e. members of the IFN pathway, with orange triangles, and IFITM3 and IFITM1 with green diamonds. The entire dataset is presented in Additional file 1: Table S1

The gating strategy for sorting a pure population of SAMHD1 negative cells is outlined in Fig. 2a. First, we sorted cells based on their morphology to remove dead cells, debris and cell doublets, which may skew subsequent analyses. Non-viable cells that cannot be identified solely by their morphology were eliminated by incubation with a viability dye, in which they appeared high in the DAPI channel. Finally, cells were sorted based on their SAMHD1 levels. As expected, only a small fraction of cells (about 7%) are SAMHD1 negative, which is consistent with data presented in Fig. 1 and with the hypothesis that only a very limited fraction of the CRISPR library will rescue SAMHD1 degradation. After sorting, we obtained 5 × 10^5^ SAMHD1 negative cells and 3 × 10^6^ SAMHD1 positive cells, which allows for a coverage of the library higher than 100X. The screen was performed with two technical replicates. After DNA extraction, sgRNA sequences in the different cell populations were amplified and deep-sequenced.

The frequency of each sgRNA within the SAMHD1 negative and positive populations was determined to calculate enrichment in the SAMHD1 negative population. sgRNAs enriched in the SAMHD1 negative fraction should target factors involved in IFN signaling, in SAMHD1 expression and stability, and may potentially target new ISGs protecting SAMHD1. In order to take into account results of all 8 sgRNAs targeting each gene in the library, we performed a gene-specific analysis with the MAGeCK tool that was developed for this purpose [28]. This method assigns a score for each gene, factoring in the combined action of all sgRNAs, the enrichment of each sgRNA, and the biological replicate of the screen. Figure 2b shows the top 20 genes from screen ranked by their-log10 MAGeCK score on the X axis. As validation of our strategy, at the very top of this list is SAMHD1 itself since sgRNAs targeting this gene should result in low protein levels of SAMHD1 (Fig. 2b, blue square). Factors necessary for IFN signaling such as the IFN receptor IFNAR1, the signaling molecules STAT1 and STAT2, and the transcription factor IRF9 are among the top scoring hits from the screen (Fig. 2b, orange triangles), further validating our strategy.

After these positive controls, IFITM3 is one of the highest hits together with poorly characterized genes such as COMMD3, EIF3L and SLC35A2. Of note, IFITM1 is also present in our list of top hits, although with a lower MAGeCK score (Fig. 2b) and IFITM2 ranks 232, with a much lower gene score (Additional file 1: Table S1). It should be pointed out, however, that because of the extensive homology between IFITMs (up to 90% at the nucleotide level), the sgRNAs used in our screen cannot completely distinguish between the different homologs. In particular, one sgRNA for IFITM1, which was much more enriched than the others, displayed perfect homology with IFITM3 (not shown).

In order to explain the IFNα-induced protection of SAMHD1 phenotype, candidate hits should be significantly induced by IFNα treatment. Thus, we compared the gene scores from our screen to IFNα induction at the mRNA level in THP1 cells using a previously published dataset [29] (Fig. 2b, Y axis). While a number of our top hits, such as COMMD3, SLC35A2 and EIF3L, are reported to be poorly induced—if at all—at the transcriptional level by IFNα [29], IFITM proteins were highly induced by IFNα in THP1 cells [29], thus appearing to be the most likely candidate hits from this screen (Fig. 2b).

### Knockout of IFITM2/3 explains most of the phenotype

**Fig. 3:**
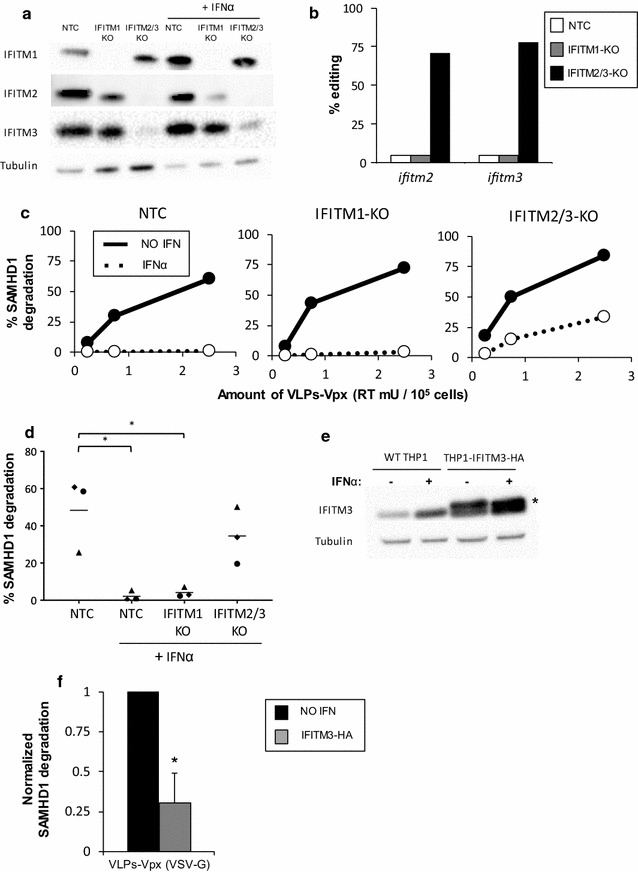
IFITMs block SAMHD1 degradation after treatment with VLPs-Vpx pseudotyped with VSV-G. **a** Knockout efficiency as measured by protein levels. THP1 were cells transduced with a lentivector coding for Cas9 and sgRNAs targeting either IFITM1 (IFITM1-KO, lanes 2 and 5) or IFITM2 and IFITM3 (IFITM2/3-KO, lanes 3 and 6) or encoding a non-targeting sgRNA (NTC, lanes 1 and 4). Cells were selected in puromycin for 2 weeks. Cells were treated for 24 h with 0 (lanes 1 through 3) or 1000 (lanes 4 through 6) U/mL IFNα and protein expression was measured by Western Blot. For IFNα treated cells, three times less lysate was loaded, in order to be able to visualize levels of IFITMs in the absence or presence of IFNα stimulation in the same exposure. Tubulin was used as a loading control. **b** Genomic DNA was extracted from THP1 NTC, IFITM1-KO or IFITM2/3-KO cells. The *ifitm2* and *ifitm3* loci were amplified using specific PCR primers with a 3’ mismatch. For each cell type, we verified that the PCR was specific by Sanger sequencing. The sequences at the two loci were compared to the reference sequence using TIDE analysis and the percentage of editing was quantified. **c** Cells were treated for 24 h with 0 or 1000 U/mL IFNα and incubated with the indicated amount of VLPs-Vpx pseudotyped with VSV-G. SAMHD1 degradation was measured by flow cytometry 16 h after treatment. One dose response experiment is shown. **d** Combined data from three independent experiments, using a viral dose within linear range. Each symbol represents an experiment. *p < 0.05 (*t* test). **e** Overexpression of IFITM3 in THP1 cells was measured by Western Blot. Cells were lysed in NP40-DOC buffer and probed for IFITM3. Tubulin was used as a loading control. The band indicated with an asterisk corresponds to the HA tagged version of IFITM3. **f** SAMHD1 degradation assays. Cells were treated for 24 h with 0 or 1000 U/mL IFNα, then incubated with the indicated amount of VLPs-Vpx pseudotyped with VSV-G for 16 h and SAMHD1 degradation was measured by flow cytometry. Combined data from three independent experiments using a viral dose within linear range. *p < 0.05 (*t* test)

To validate the results of our screen, we generated CRISPR knockout THP1 cells for specific IFITM proteins. Achieving knockout specificity for IFITMs is technically challenging, as previous studies have pointed out [25, 30], in particular for IFITM2 and IFITM3, which share 90% sequence identity at the nucleotide level. Therefore, we selected one sgRNA that specifically targets IFITM1 (12 and 13 nucleotide changes from IFITM2 and IFITM3, respectively) and one sgRNA that targets both IFITM2 and IFITM3 as it has perfect homology for both loci in THP1 cells, but differs by three nucleotides from IFITM1. A non-targeting control (NTC) sgRNA was included as a negative control. These sgRNAs were cloned into the Cas9-expressing lentiviral vector, and THP1 cells were transduced and selected by puromycin treatment. We first attempted to generate clonal knockout cell lines, but heterogeneity of the cell line, a known issue with THP1 cells [31], caused significant clone-to-clone variability and complicated interpretation of the results (not shown). Instead, we used pools of knockout cells, in which, after puromycin selection, levels of IFITM expression were greatly reduced, as measured by Western Blot (Fig. 3a). We observed that the IFITM1 sgRNA appeared to be specific, as it knocked-out almost entirely the expression of IFITM1 without significantly altering levels of IFITM2 and IFITM3. The sgRNA targeting IFITM2/3 led to low levels of expression of both IFITM3 and IFITM2, even in presence of IFNα, but left IFITM1 intact. Antibody specificity for IFITM2 vs IFITM3 is known to be difficult to achieve. Thus, to confirm our Western Blot results, we designed primers specific for the *ifitm2* and *ifitm3* loci (details in the Methods section), that were used for PCR amplification and sequencing of the CRISPR lesions present in THP1 knockout cells (Fig. 3b). Using TIDE analysis, a tool specifically designed to assess the percentage of cells edited by sgRNAs in heterogeneous populations [32], we determined that IFITM2/3 knockout cells are highly edited for *ifitm2* (71%) and *ifitm3* (78%), while NTC and IFITM1 knockout cells showed no editing at these loci. These results confirm that the sgRNA has specificity for both IFITM2 and IFITM3, as the IFITM2/3 knockout cells are edited at both the *ifitm2* and *ifitm3* loci, but not at the *ifitm1* locus.

To ask if IFITM knockout rescues SAMHD1 degradation, knockout cell lines were treated with IFNα and with Vpx, as described previously, and SAMHD1 degradation was measured. We observed that knockout of IFITM2/3, but not IFITM1, led to a significant rescue of SAMHD1 degradation in presence of IFNα. In the experiment presented in Fig. 3c, up to 34% of IFITM2/3 knockout cells (vs < 1% in NTC cells) had SAMHD1 degraded in the presence of IFNα. Averaged over three independent experiments, we observed that knocking out IFITM2/3 significantly rescued SAMHD1 degradation (Fig. 3d). These results suggest that IFITM3 and/or IFITM2, but not IFITM1, play a major role in inhibiting SAMHD1 degradation by VLPs-Vpx.

Since IFITM3 was the top ISG (apart from positive controls) enriched in our initial screen, we also generated a stable THP1 cell line over-expressing an HA-tagged version of IFITM3 (IFTIM3-HA), in which the levels of IFITM3 are within fivefold of those observed after IFNα treatment, as measured by Western Blot (Fig. 3e). We found that IFITM3-HA over-expression potently inhibited the ability of VSV-G pseudotyped VLPs-Vpx to degrade SAMHD1 in the absence of IFNα: averaged over 3 experiments, we observed a fourfold reduction in SAMHD1 degradation in IFITM3 expressing cells, compared to wild-type THP1 (Fig. 3f). The results of both knockout and over-expression experiments thus show that IFITM2/3 inhibit SAMHD1 degradation in THP1 cells.

### IFNα protection of SAMHD1 is envelope-dependent and affects entry of Vpx bearing VLPs into cells

**Fig. 4:**
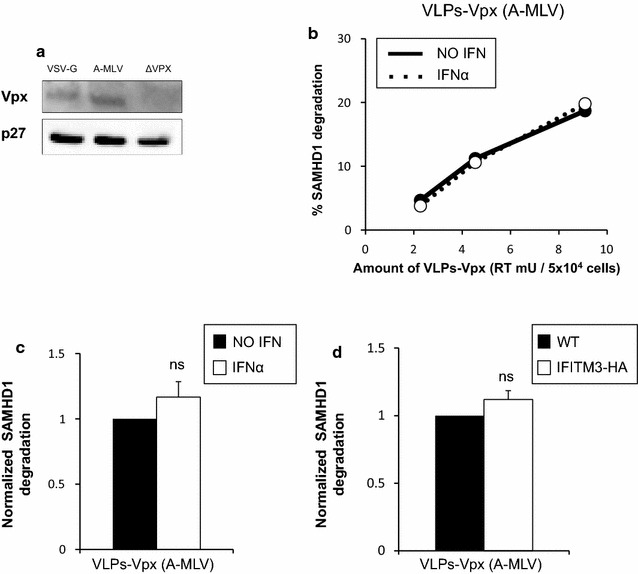
IFN α does not block SAMHD1 degradation in THP1 cells when VLPs-Vpx are pseudotyped with A-MLV. **a** Levels of Vpx incorporation in VLPs pseudotyped with different envelopes. 250 RT mU of VLPs-Vpx pseudotyped with either VSV-G (left lane) or A-MLV (center lane) were loaded on a gel and levels of Vpx were probed by Western Blot. 250 RT mU of VLPs-∆*vpx* pseudotyped with VSV-G (rigth lane) were used as a negative control. 50 RT mU of the same VLPs preparations were loaded and probed for p27 as a loading control. **b** THP1 cells were treated for 24 h with 0 (solid lines) or 1000 (dotted lines) U/mL of IFNα, then with the indicated amount of VLPs–Vpx pseudotyped with A-MLV (as determined by RT activity). Cells were treated with the indicated amounts of VLPs-Vpx and SAMHD1 degradation was measured by flow cytometry 16 h later. One representative experiment is shown. **c** Combined data from three independent experiments for the effect of IFNα (white bar), using a viral dose within linear range. n = 3; *ns* non significant (*t* test). **d** Combined data from three independent experiments for the effect of IFITM3 overexpression (white bar), using a viral dose within linear range. n = 3; *ns* non significant (*t* test)

We hypothesized that IFITMs may act on SAMHD1 degradation by inhibiting VSV-G endosomal entry, as this envelope was used to pseudotype the Virus-Like Particles containing Vpx. Importantly, while IFITM3 restricts entry of VSV [6] and of lentiviral vectors pseudotyped with the VSV-G envelope [33], it has no effect on entry mediated by the Murine Leukemia Virus amphotropic envelope (A-MLV) [4]. Unlike VSV, MLV is thought to fuse mainly at the plasma membrane [34], although some reports suggest that it uses caveolae-mediated endocytosis [35, 36]. Thus, we compared VLPs-Vpx pseudotyped with VSV-G, which had been used in Fig. 1 and in previous studies [10, 12, 22, 27], with VLPs-Vpx pseudotyped with the Murine Leukemia Virus amphotropic envelope (A-MLV). We normalized the amounts of VLPs-Vpx used based on RT activity, and verified that the different VLPs packaged roughly similar levels of Vpx (Fig. 4a). When A-MLV was used instead of VSV-G, about 6 times more VLPs were required in order to achieve similar levels of SAMHD1 degradation, indicating that this envelope may be less fusogenic (Fig. 1b, ~ 1.5 RT mU of VLPs VSV-G lead to 20% degradation compared to 9 RT mU of VLPs A-MLV, Fig. 4b). Strikingly, we observed that while A-MLV env-pseudotyped Vpx-containing particles were able to cause degradation of SAMHD1 in the absence of IFNα (Fig. 4b, black lines), IFNα treatment had no effect on Vpx-mediated SAMHD1 degradation (Fig. 4b, dashed line). Averaged over three independent experiments, we found that there is no significant difference in SAMHD1 degradation by VLPs-Vpx particles pseudotyped with A-MLV (Fig. 4c), in contrast to VLPs-Vpx particles pseudotyped with VSV-G (Fig. 1c). IFITM3-HA over-expression also had no effect on SAMHD1 degradation when the A-MLV env was used (Fig. 4d), consistent with previous reports [4]. These results provide further evidence that, rather than directly acting on the SAMHD1/Vpx interface or on Vpx itself, IFNα likely acts at the earlier stage of Vpx delivery by VLPs.

In order to initiate degradation of SAMHD1, Vpx must be released from incoming virions, enter the nucleus [12] and engage the DCAF4/DDB1/Cul4a complex [15, 16]. Thus, monitoring SAMHD1 degradation is an indirect measure of the effects of IFNα and IFITMs on blocking release of viral components first into the cytoplasm. To show more directly that IFNα affects an early step of Vpx delivery into cells, we used the Vpr-β-lactamase (Vpr-BlaM) assay, which measures entry of viral cores into the cytoplasm specifically, without taking into account capture of viral particles in endosomes.

**Fig. 5:**
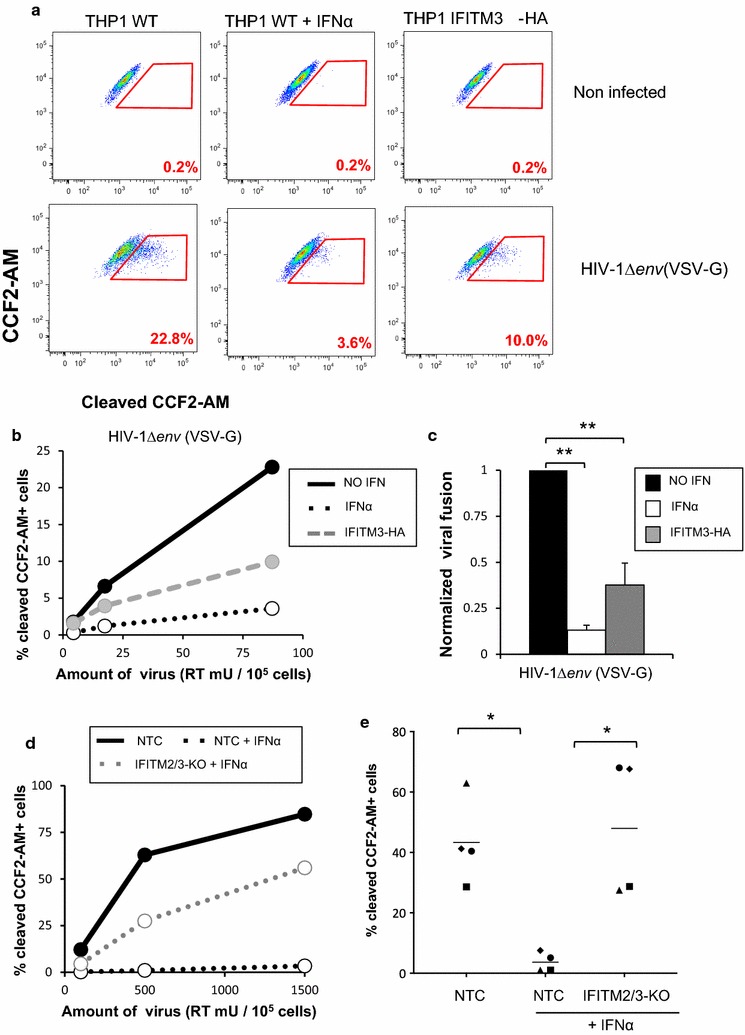
IFN α treatment and IFITMs directly block viral fusion. **a** THP1 wild-type or overexpressing IFITM3-HA were treated for 24 h with 0 or 1000 U/mL IFNα for 24 h, and then infected with the indicated amount of HIV-1Δ*env*(VSV-G) packaging the Vpr-β-lactamase fusion protein for 3 h. Cells were then incubated with the fluorescent CCF2-AM substrate for 2 h, fixed, and acquired by flow cytometry immediately. Representative flow cytometry plots are shown. **b** Representative dose response experiment. Solid black line: wild-type THP1 cells, dashed black line: wild-type THP1 treated with IFNα, grey dashed line: THP1 cells overexpressing IFITM3. **c** Combined data from three independent experiments, using a viral dose within linear range. **p < 0.01 (paired *t* test). **d** THP1 cells wild-type (in black) or knockout for IFITM2/3 (in grey) were treated with IFNα (dashed lines) or untreated (solid lines) and infected with the indicated amount or HIV∆*env*(VSV-G). One representative dose response experiment is shown. **e** Combined data from four independent experiments, using a viral dose within linear range. Each symbol represents data from one experiment. **p < 0.01 (paired *t* test)

We produced HIV∆*env* pseudotyped with VSV-G (HIVΔ*env*(VSV-G)) packaging Vpr-BlaM, and infected THP1 cells. Representative flow cytometry data and a dose response experiment are shown in Fig. 5a, b, respectively. IFITM3-HA over-expression significantly restricted VSV-G-mediated entry by about threefold (Fig. 5c), consistent with its effect on Vpx delivery (Fig. 3b). IFNα had an even stronger effect, inhibiting VSV-G entry by about eightfold. This result further demonstrates that IFNα treatment and IFITM3 expression are able to restrict entry of viral particles pseudotyped with VSV-G, excluding other indirect effects on Vpx or SAMHD1. Similar to our results using SAMHD1 degradation experiments (Fig. 3), knocking-out IFITM2/3 rescued VSV-G fusion in the presence of IFNα (Fig. 5d): the inhibition by IFNα decreased from roughly 30-fold to 1.5-fold when IFITM2/3 were knocked-out even though we used pools of cells where the knockout efficiency was around 75% (Fig. 3b). Averaged over four experiments, IFITM2/3 knockout significantly rescued entry of VSV-G pseudotyped particles into cells (Fig. 5e), demonstrating that IFITM2/3 indeed contribute to the IFNα entry block to VSV-G entry. Although we cannot exclude a role for other ISGs, we conclude that the main mechanism by which IFNα protects SAMHD1 from degradation is by inducing expression of IFITMs that block VSV-G-mediated entry of VLPs-Vpx.

### IFNα and IFITMs impose a stronger block on VSV-G-pseudotyped viruses than on wild-type HIV-1

**Fig. 6:**
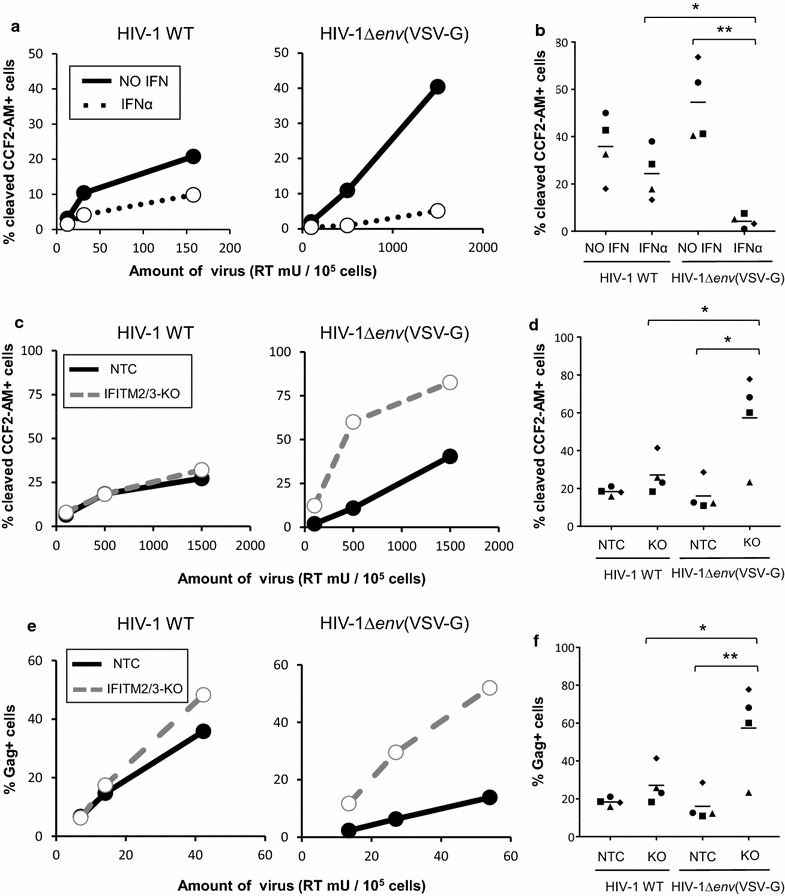
VSV-G pseudotyping leads to stronger restriction by IFNα and IFITM2/3. **a**, **b** THP1 wild-type or overexpressing IFITM3-HA were treated for 24 h with 0 or 1000 U/mL IFNα for 24 h, and then infected with the indicated amounts of either HIV-1 WT (**a**, left panel) or HIV-1Δ*env*(VSV-G) (**a**, right panel) packaging the Vpr-β-lactamase fusion protein for 3 h. Cells were then incubated with the fluorescent CCF2-AM substrate for 2 h, fixed, and acquired by flow cytometry immediately. **a** Representative dose response experiment. **b** Combined data from four independent experiments, using a viral dose within linear range. Each symbol representing data from one experiment. *p < 0.05; **p < 0.01 (*t* test). **c**, **d** THP1 cells transduced with a Non-Targeting Control (NTC) sgRNA or with an sgRNA targeting both IFITM2 and IFITM3 (IFITM2/3) were infected, in the absence of IFNα, with HIV WT (**c**, left panel) or HIV-1Δ*env*(VSV-G) (**c**, right panel) packaging Vpr BlaM for 3 h. Viral entry was measured as in panel **a**. **c** One representative dose response experiment. **d** Combined data from four independent experiments, using a viral dose within linear range. Each symbol represents one experiment. *p < 0.05 (*t* test). **e**, **f** THP1 cells transduced with a non-targeting sgRNA (NTC) or with a sgRNA against IFITM2 and IFITM3 (IFITM2/3) were infected, in absence of IFNα, with HIV WT (**e**, left panel) or HIV-1Δ*env*(VSV-G) (**e**, right panel). After 24 h, viral input was washed, and cells were cultured in presence of 1 μM of the entry inhibitor T-20, to prevent subsequence rounds of infection. The percentage of Gag positive cells were measured by flow cytometry at 48 h p.i. E: One representative dose response experiment. **f** Combined data from four independent experiments, using a viral dose within linear range. Each symbol representing data from one experiment. Each symbol represents one experiment. *p < 0.05, **p < 0.01 (*t* test)

IFNα treatment, in part through the action of IFITMs, blocks entry of VSV-G pseudotyped viral particles. Because entry with the VSV-G envelope may differ from how HIV-1 enters cells we compared the magnitude of this block to the one imposed on HIV-1’s own CXCR4-tropic envelope. Importantly, these two envelopes have different mechanisms for viral entry: while VSV-G-mediated entry occurs through pH-dependent fusion in the endosomes [37], productive entry of HIV-1 into its target cells likely happens by pH-independent fusion at the plasma membrane, although this remains controversial [38, 39]. Thus, we reasoned that the site of viral entry might dictate the sensitivity to IFNα and to IFITMs and affect lentiviruses bearing the HIV-1 or VSV-G envelopes differentially. To test this hypothesis, we directly compared the amount of IFNα block to HIV-1 that either contained its natural env  or was pseudotyped with VSV-G in the entry assay described previously (Fig. 5). Strikingly, while we observed stronger IFNα block on VSV-G-mediated entry than for HIV-1 env-mediated entry  (24-fold vs 1.5-fold on average, respectively; Fig. 6a, b). Similarly, knockout of basal levels of IFITM2/3 (in absence of IFNα) had a significant impact on VSV-G-mediated entry, but much less on entry mediated by HIV-1 Env (fourfold vs 1.5-fold on average, Fig. 6c, d). In both cases, statistical analysis indicated that infection by VSV-G pseudotypes was significantly different than HIV-1 wt either in the presence of IFNα or in untreated cells lacking IFITM2 and IFITM3. These results suggest that VSV-G pseudotyping may expose the virus to a different subset of restriction factors that restrict viral fusion, including IFITMs, potentially by diverting HIV-1 from its natural entry pathway. These results are consistent with previous observations linking the route of HIV-1 entry and IFITM restriction [25].

To confirm that this difference in entry translates into different levels of infection in cell cultures, we performed single cycle infection assays. THP1 cells transduced with lentivectors encoding Non-Targeting Control (NTC) or IFITM2/3-targeting sgRNAs were infected with either HIV-1∆*env*(VSV-G), which is only able to perform one cycle of infection, or with wild-type HIV-1. In order to ensure that the latter virus only performs one round of infection, the viral input was washed after 24 h and cells were treated with T-20, a peptide inhibiting membrane fusion of HIV-1 [40]. These experiments were performed in absence of IFNα with the rationale that other ISGs potently inhibiting HIV in THP1 cells, such as MxB could mask the effect of IFITMs [29]. Notably, IFITMs are significantly expressed at basal levels (Fig. 3a). Infection was monitored by measuring intracellular Gag levels 48 h post-infection. Consistent with our entry results, we observed that IFITM2/3 knockout greatly enhanced infection by HIV-1∆*env*(VSV-G), but had a much less pronounced effect on wild-type HIV-1 infection (Fig. 6e, f). Taken together, these results suggest that basal levels of IFITM2 and IFITM3 expression inhibit VSV-G-mediated entry, but much less HIV-1 entry. Furthermore, we conclude that VSV-G-pseudotyping, although achieving higher rates of infection thanks to a higher envelope fusogenicity, changes the sensitivity of HIV-1 to an IFNα entry block by increasing sensitivity to IFITM-mediated restriction.

## Discussion

### A CRISPR screen for IFNα-induced factors that protect SAMHD1 from degradation

A flow cytometry-based CRISPR knockout screen approach identified IFITMs as being the most predominant ISGs underlying the protection of SAMHD1 from degradation after transduction with VSV-G-pseudotyped VLPs containing Vpx [22]. As the VSV-G used to pseudotype the VLPs delivering Vpx mediates entry into endosomes [37], where IFITMs (particularly IFITM3) are thought to act, our results argue that IFNα protects SAMHD1 from degradation by blocking viral entry of VLPs delivering Vpx into cells. The method described here that assays viral entry/fusion events by monitoring Vpx-mediated degradation of SAMHD1, could also be applied more generally to discover new genes affecting entry mediated by different envelope proteins.

### IFITMs directly contribute to the effect of IFNα on SAMHD1 degradation by VLPs-Vpx

Because of high sequence homology, targeting IFITM3 without affecting levels of IFITM2 is challenging, as described in previous studies [25, 30]. Thus, we could not formally demonstrate if IFITM2 or IFITM3 act synergistically, or alone in this phenotype. Nonetheless, we did show that IFITM3-HA overexpression is sufficient to inhibit degradation of SAMHD1 by VLPs-Vpx (Fig. 3). Although one previous study observed only a minor effect of IFNα treatment on viral entry of VSV-G-pseudotyped lentivectors using the Vpr-BlaM assay [22], our eightfold IFNα block of VSV-G entry (Fig. 5), together with the fact that IFNα has no effect when an alternate delivery method was used (Fig. 4), strongly supports the idea that IFITMs block delivery of Vpx into the cytoplasm of IFNα-treated cells. Therefore, the SAMHD1 protection phenotype described in the literature [22] can be explained by inhibition of Vpx delivery by IFITMs. Finally, other factors, such as COMMD3 or EIF3L also emerged as top hits from our screen (Fig. 2), and although they were minimally induced (if at all) by IFNα in THP1 cells, they may contribute to early events in VSV-G-mediated entry into cells.

### VSV-G pseudotyping changes HIV-1 sensitivity to IFNα and IFITMs

In addition to blocking endosomal entry of VSV [6], IFITMs are also described to inhibit HIV-1 entry when expressed in target cells [41]. IFITM restriction in target cells may rely on a different mechanism than when IFITMs are expressed in producer cells. In addition to the role of IFITMs that are packaged into budding virions and restrict infection in target cells [33, 42, 43], IFITMs may, when expressed in target cells, also inhibit the hemi-fusion process [44, 45], and/or deregulate cholesterol homeostasis, resulting in reduced membrane fusion [46].

Changing the entry pathway used by HIV-1 by VSV-G pseudotyping directs membrane fusion to the endosomes instead of at the plasma membrane, and may influence HIV-1 sensitivity to ISGs, and to IFITMs in particular. Consistent with this idea, we observed that VSV-G pseudotypes are hyper sensitive to IFNα and IFITMs (Fig. 6). Moreover, the effect of IFITM3-HA on VSV-G-mediated entry did not completely recapitulate the magnitude of the IFNα block (Fig. 5), suggesting that other ISGs may play an additional role.

A recent report demonstrating that HIV-1 Transmitter/Founder strains are initially resistant to IFITM3, but become sensitive after viral escape from neutralizing antibodies [25]. Although it is important to note that the subcellular compartment in which HIV-1 fuses and enter cells leading to productive infection remains controversial, and may depend on the cell type used [38, 39], one likely interpretation of our results is that in THP1 cells, HIV-1 fuses predominantly at the plasma membrane, rather than in endosomal vesicles where IFITM3 and other ISGs may act. VSV-G pseudotyping, by re-routing HIV-1 fusion to endosomes, may expose the virus to a different set of restriction factors. Our data suggest that HIV-1 strains that have evolved to fuse at the plasma membrane would avoid a potentially more restrictive environment imposed by IFITM2/3. The endosomal compartment may indeed represent a less productive route for HIV-1 entry, as has been suggested by a study in which a dominant negative variant of dynamin blocked endocytosis but had no effect on infection levels in T cells [47]. Importantly, VSV-G pseudotyping may not only affect viral entry, but also later steps in the viral life cycle, such as uncoating [48] or nuclear import and integration [49]. Thus, such considerations should be taken into account in studies that use VSV-G pseudotyping, as it may influence the antiviral factors that HIV-1 may be accessible to.

## Conclusions

In this work, we used a CRISPR-Cas9 knockout flow cytometry screen to determine factor(s) are responsible for the block to SAMHD1 degradation in IFNα treated cells when Vpx is delivered via VSV-G pseudotyped viral particles. We identified IFITMs as the major factors involved in this phenomenon. Our results suggest that IFNα acts on Vpx delivery due to the effects of IFITMs on VSV-G-mediated entry/fusion rather than on the SAMHD1 degradation process itself. Indeed, we show that IFNα and IFITM2/3 have a much greater effect on entry of HIV-1 pseudotyped with VSV-G compared to HIV-1 bearing a CXCR4-tropic envelope. Thus, using VSV-G pseudotypes to study HIV restriction factors can be misleading because these pseudotypes may be exposed to antiviral factors that do not normally affect HIV-1. Finally, the CRISPR knockout screening approach we describe here could also be used to identify restriction factors specific for different viral envelopes using Vpx-mediated SAMHD1 degradation as an easy readout to screen for viral entry.

## Material and methods

### Cells, plasmids and viruses

THP1 cells were grown in RPMI 1640, supplemented with 10% Fetal Bovine Serum (FBS) and penicillin/streptomycin (100 μg/mL; Gibco #15140-122). HEK-293T cells were grown in Dulbecco modified Eagle medium (DMEM) supplemented with 10% FBS and penicillin/streptomycin. The SIV3 + , SIV3∆*vpx* and Vpr-β-lactamase plasmids are kind gifts from Olivier Schwartz [10, 11, 27, 50]. The pHIV-dTomato plasmid was obtained through Addgene (#21374). The sgRNA library was synthesized in vitro (Twist Bioscience) and cloned in the lentiCRISPRv2 lentiviral backbone, and was a gift from Feng Zhang (Addgene plasmid # 52961). pMD2.G and psPAX2 were gifts from Didier Trono (Addgene plasmids #12259/12260). VLPs-Vpx were produced by transfecting HEK293T cells with SIV_MAC_ Gag/Pol (SIV3 + plasmid or SIV3Δ*vpx*) and VSV-G (pMD2.G plasmid) or A-MLV (A-MLV Env plasmid) at a 2.5: 1 ratio, and using 3 μL of TransIT LT1 (#MIR2305, Mirus) per μg of DNA. HIV-1 Vpr-β-lactamase viruses were produced by co-transfecting either HIV-1 proviral wild-type plasmid (pLAI) and Vpr-β-lactamase at a 3:1 ratio, or HIV-1∆*env* (pLAI∆*env*), VSV-G, and Vpr-β-lactamase at a 3:1:1 ratio. IFITM3-HA lentivector particles were made by co-transfecting the pHIV-dTomato-IFITM3-HA plasmid together with HIV-1 Gag/Pol (psPAX2 plasmid) and VSV-G at a 2:1.5:1 ratio. Lentiviral vector preparations containing the ISG library were made using the lentiCRISPRv2_ISG library assembly together with psPAX2 and VSV-G at a 2:1.5:1 ratio. For all virus and VLP production, supernatants were harvested 48–72 h post transfection, clarified by centrifugation, passed through a 0.22 μm filter and concentrated by ultracentrifugation (90 min at 90,000 g). All transduction and infection experiments were carried out in presence of 20 μg/mL of DEAE-Dextran (Sigma; # D9885). The following reagents were obtained through the NIH AIDS Reagent Program, Division of AIDS, NIAID, NIH: T-20,(Enfuvirtide); SV-A-MLV-Env plasmid from Dr. Nathaniel Landau and Dr. Dan Littman [51].

### SAMHD1 degradation assay

5x10^4^ THP1 cells were plated in 96-well plates and treated with 1000 U/mL universal type I IFNα (#11200-2, PBL) or IFNγ (#11500-2, PBL) for 24 h. The next day the cells were treated with the indicated amount of VLPs-Vpx for 16 h. Cells were washed in PBS, and incubated for 15 min at room temperature with the Live/Dead Fixable Blue Dead Cell Stain Kit following instructions provided by the manufacturer (#L34962, ThermoFisher), then fixed in 4% formaldehyde solution (diluted in PBS from a 37% solution, Sigma #252549) for 5 min, and then permeabilized using PBS-Triton X-100 (Sigma, #X100) 0.5% for 15 min. Cells were then stained on ice for 30 min with anti-SAMHD1 antibody (clone I19-18, Millipore #MABF933,) diluted to 0.5 μg/mL in PBS 1% BSA, washed in PBS, and incubated on ice for 30 min with the Goat anti-mouse Alexa 488 secondary antibody (#A-11001, Thermo Fisher), diluted to 4 μg/mL in PBS 1% BSA. Flow cytometry data were acquired using a CANTO-II flow cytometer (BD).

### Flow cytometry CRISPR screen

The screening experiment was performed with two technical replicates. 5x10^7^ THP1 cells were transduced with lentiviral vectors containing the lentiCRISPRv2_ISG library. After 2 weeks of puromycin selection (0.5 μg/mL, #P8833, Sigma), cells were treated with 1000 U/mL of universal type I IFNα for 24 h and incubated with VLPs-Vpx for 16 h. Cells were washed in PBS and incubated for 30 min at room temperature with the Live/Dead Fixable Blue Dead Cell Stain Kit following instructions provided by the manufacturer. Cells were washed in PBS and fixed with 1% PFA for 15 min. The fixation process was stopped by addition of 0.2 M Glycine. Cells were washed in PBS, and permeabilized using PBS/Triton X100 0.5% for 15 min. Cells were washed with PBS, stained on ice for 60 min with anti-SAMHD1 antibody diluted at 0.5 μg/mL in PBS 1% BSA, washed in PBS, and incubated on ice for 45 min with the Goat anti-mouse Alexa 488 secondary antibody diluted to 4 μg/mL in PBS 1% BSA. Cells were washed and resuspended in sorting buffer (PBS 2% FBS, 25 mM Hepes, 5 mM EDTA) and filtered. 5 × 10^5^ SAMHD1 negative and 3 × 10^6^ SAMHD1 positive cells were then sorted using an ARIA-II flow cytometer (BD), in polypropylene tubes previously coated with PBS 4% BSA. Cells were pelleted and lysed in 300 μL of chromatin immunoprecipitation buffer (0.1% SDS, 10 mM EDTA, 20 mM EGTA, 300 mM NaCl, 10 mM Tris HCl pH 8.1). 3 μL of proteinase K (#19133, Qiagen) and 3 μL of RNAse A (10 μg/mL, #R4642-10MG, Sigma) were added to the lysates and cells were incubated at 65 °C for 16 h to reverse crosslinking of protein and DNA. DNA was extracted using phenol chloroform (#P2069-100 ML, Sigma), precipitated, washed in 70% ethanol, and resuspended in RNAse free water. sgRNAs were amplified from genomic DNA with a maximum of 2 μg of template DNA per reaction using the Herculase II Fusion DNA Polymerase kit (Agilent, #600679). A first PCR was carried out using the following primers: forward gagggcctatttcccatgattccttca and reverse ctgctgtccctgtaataaacccg. Round 1 PCR products were cleaned up using the QIAquick PCR purification kit (Qiagen, #28106) and used as a template for the round 2 PCR, that allows barcoding and addition of the sequencing adapters, using a common reverse primer caagcagaagacggcatacgagatgtgactggagttcagacgtgtgctcttccgatcttgccactttttcaagttgataacggact coupled with a unique barcoding forward primer for each sample, here: F1 aatgatacggcgaccaccgagatctacactctttccctacacgacgctcttccgatctatctcgcgtacgtcttgtggaaaggacgaaacaccg and F2 aatgatacggcgaccaccgagatctacactctttccctacacgacgctcttccgatctactacagtgtcttgtggaaaggacgaaacaccg. PCR products were cleaned up and size selected using the Agencourt Ampure XP beads kit (Beckman Coulter, # A63880), using a two-step purification with 1:1.2 and 1:1.5 DNA to beads ratios. Samples were quantified using the QUBIT dsDNA HS Assay Kit (Thermo Fisher Q32854), pooled at a 2 nM concentration and submitted for sequencing on one HiSeq2500 (Illumina) lane, using the Rapid Mode (60 cycles).

### Statistical analysis

sgRNA enrichment was analyzed using the Bioconductor package edgeR [52] and gene-level analysis was performed using MAGeCK [28]. The IFNα induction data was generated using a dataset (GSE46599) published by Goujon et al. [29] and re-analyzed using the lumi [53] and limma [54] Bioconductor packages. When several probes were used for a single genes, fold induction values were averaged.

### Reverse-transcriptase activity assay

Viral stocks were quantified using an RT activity assay described before [55]. Briefly, viral supernatants were lysed in 2X lysis buffer (0.25% Triton X-100, 50 mM KCl, 100 mM Tris HCl, glycerol 40%) in the presence of 4U RNAse inhibitor (Fermentas, #EO0382). qRT-PCR reactions were prepared following instructions from the Takyon Rox SYBR MasterMix dTTP Blue kit (Eurogentec, #UF-RSMT-B0101), with MS2 RNA used as a template (Roche, #10165948001) and the following primers: tcctgctcaacttcctgtcgag and cacaggtcaaacctcctaggaatg. qRT-PCR was performed using a ABI QuantStudio5 Real Time PCR machine. Reactions were performed in duplicates, and titers were calculated using a standard curve made with a virus stock of previously determined RT units.

### Vpr-β-lactamase assays

Viral entry was assayed with a protocol adapted from Cavrois et al. [50]. Briefly, indicated amounts of virus containing the Vpr-β-lactamase fusion protein were used to infect 10^5^ THP1 cells. After 3 h, cells were washed in cold CO_2_-independent media (Invitrogen), without FBS, resuspended in CO_2_-independent media supplemented with 10% FBS, and incubated with the CCF2-AM substrate following the instructions provided by the manufacturer (LiveBlazer FRET—B/G loading kit, #K1023, Invitrogen), in the presence of 1.8 mM Probenecid (#P8761-25G, Sigma), for 2 h at room temperature in the dark. Cells were washed three times in cold CO_2_-independent media, then once in PBS, and finally fixed with 4% formaldehyde for 10 min. Fluorescence of the CCF2-AM substrate was immediately measured by flow cytometry on a Canto-II (BD) using the AmCyan and Pacific-Blue channels.

### Generation of cell lines overexpressing IFITMs or with IFITM knockout

RNA was extracted from 10^6^ THP1 cells using the Rneasy plus mini Kit (#74134, Qiagen). RT-PCR was performed to amplify IFITM3 cDNA and add a C-terminal HA-tag and restriction sites using the SuperScript^®^ III One-Step RT-PCR System with Platinum Taq DNA Polymerase kit (#12574018, Invitrogen) and the following primers: gatctctagaatcgatatgaatcacactgtccaaacct and gatcggatccggtaccctaagcgtaatctggaacatcgtatgggtatccataggcctggaagatca. IFITM3-HA was then cloned into the pHIV-dTomato backbone using the XbaI/BamHI restriction sites, and clones were verified by sequencing. THP1 cells were spinoculated with lentivectors encoding IFITM3-HA, and overexpression was verified by monitoring dTomato levels. Cells were harvested and lysed in the NP40-DOC lysis buffer (NP40 1%, deoxycholate 0.2%, NaCl 120 mM, Tris 20 mM) and IFITM3 levels were also monitored by Western Blot analysis (#11714-1-AP, Proteintech; 1/1000). sgRNAs targeting IFITM1 or IFITM2/3 were cloned in the lentiCRISPRv2 backbone by BsmBI restriction of the backbone, annealing of sense and antisense oligos (5 min at 95 °C) and ligation. The following oligos were used (the 20 bp-gene targeting sequence in all caps): IFITM1 sense caccgCAGAGCCGAATACCAGTAAC and antisense aaacGTTACT GGTATTCGGCTCTGc; IFITM2/3 sense caccgGTGGATCACGGTGGACGTCG and antisense aaacCGACGTCCACCGTGATCCACc; NTC sense caccgACGGAGGCTAAGCGTCGCAA and antisense aaacTTGCGACGCTTAGCCTCCGTc. Lentiviral particles were produced as indicated before and used to transduce THP1 cells. After 1-2 weeks of puromycin selection (1 μg/mL, #P8833, Sigma), genomic DNA was extracted using the QuickExtract kit (Lucigen, QE09050) by resuspending cells in 100 μL of the solution, and by denaturing for 20 min at 60 °C and 20 min at 95 °C. The following primers, specific for each *ifitm* locus due to a mismatch at the 3’ nucleotide, were used for locus specific amplification: IFITM2-F aagaggaaactgttgagaaaacgg, IFITM2-R cgtgtgaggataaagggctgatg, IFITM3-F accatcccagtaacccgaccg, IFITM3-R gctgatacaggactcggctcc. Amplicons were sequenced and the percentage of editing was quantified using TIDE analysis [32] (https://tide-calculator.nki.nl/). Knockout was also verified by Western Blot using NP40-DOC as the lysis buffer and antibodies specific for IFITM1 (#60074-1-Ig, Proteintech; 1/1000), IFITM2 (#66137-1-Ig, Proteintech; 1/1000), IFITM3 (#11714-1-AP, Proteintech; 1/1000).

## Single cycle infection assays

10^5^ cells were infected with HIV-1 WT or HIV-1Δ*env*(VSV-G) with the indicated amounts of virus. After 24 h, cells were washed in PBS and the T-20 entry inhibitor (NIH AIDS reagent program, #12732) was added at a 1 μM concentration to prevent subsequent rounds of infection. Intracellular Gag levels were measured by flow cytometry 36 h post infection using KC57-FITC (Beckman Coulter #664665).

## Additional file

**Additional file 1: Table S1.** Complete MAGeCK analysis. For each gene present in the knockout ISG library, we indicate the MAGeCK gene score, rank, and the associated *p* value as determined by MAGeCK. Values of IFNα induction based on Goujon et al. [29] are also shown
